# Retraction Note: Evaluating the impact of school-based influenza vaccination programme on absenteeism and outbreaks at schools in Hong Kong: a retrospective cohort study protocol

**DOI:** 10.1186/s41043-026-01435-2

**Published:** 2026-08-19

**Authors:** Chuhan Miao, Qingyang Lu, Yuqian Wu, Jianxun He

**Affiliations:** 1 School of Public Health, Li Ka Shing Faculty of Medicine, The University of Hong Kong, No.5 Sassoon Road, Pokfulam, Hong Kong SAR China; 2 Department of Neurosurgery, Gansu Provincial Maternity and Child Care Hospital, No. 999 Mogao Avenue, Lanzhou, Gansu China

**Retraction Note: Journal of Health**,** Population and Nutrition (2024) 43:62**

10.1186/s41043-024-00561-z

The Editors-in-Chief have retracted this article.

After publication, concerns were raised regarding the ethics approval (IRB No: UW 17–111). The Hospital Authority Hong Kong West Cluster IRB Committee has confirmed that the IRB approval was not approved for the study by the authors listed in this publication.

Author Jianxun He agrees with this retraction. Author Chuhan Miao and Yuqian Wu disagree with this retraction. Author Qingyang Lu has not responded to correspondence regarding this retraction.

